# The alarms should no longer be ignored: survey of the demand, capacity and provision of adult community eating disorder services in England and Scotland before COVID-19

**DOI:** 10.1192/bjb.2023.57

**Published:** 2024-08

**Authors:** David Viljoen, Emily King, Sophie Harris, Jonathan Hollyman, Kate Costello, Eimear Galvin, Melissa Stock, Ulrike Schmidt, James Downs, Murali Sekar, Ciaran Newell, Sam Clark-Stone, Amy Wicksteed, Caroline Foster, Francesca Battisti, Laura Williams, Roshan Jones, Sarah Beglin, Stephen Anderson, Thuthirna Jebarsan, Viviane Ghuys, Agnes Ayton

**Affiliations:** 1 Oxford Health NHS Foundation Trust (OHFT), Oxford, UK; 2 Ellern Mede Ridgeway and Ellern Mede Barnet, London, UK; 3 Support to Recovery in Disordered Eating Service for 18–25s, Central and North West London NHS Foundation Trust, London, UK; 4 Salomons Institute for Applied Psychology, Canterbury Christ Church University, Canterbury, UK; 5 University of Edinburgh, Lothian NHS, Edinburgh, UK; 6 Oxford Institute of Clinical Psychology Training and Research, Oxford University and Oxford Health NHS Foundation Trust, Oxford, UK; 7 Hertfordshire Community Eating Disorders Service, Hertfordshire Partnership University NHS Foundation Trust, Hatfield, UK; 8 Institute of Psychiatry, Psychology and Neuroscience, King's College London, London, UK; 9 Royal College of Psychiatrists, London, UK; 10 Priory Group, Chelmsford, UK; 11 Dorset Eating Disorders Service, Dorset Healthcare University NHS Foundation Trust, Poole, UK; 12 Bournemouth University, Poole, UK; 13 Eating Disorder Service, Gloucestershire Health and Care NHS Foundation Trust, Brockworth, UK; 14 Sheffield Eating Disorder Service, Sheffield Health and Social Care NHS Foundation Trust, Sheffield, UK; 15 Adult Eating Disorder Service, Surrey and Borders Partnership NHS Foundation Trust, Leatherhead, UK; 16 Wiltshire Community Eating Disorders Service, OHFT, Oxford, UK; 17 Oxford Child and Adolescent Eating Disorder Service, Oxford Health NHS Foundation Trust, Oxford, UK; 18 Adult Eating Disorder Service, Cambridgeshire and Peterborough NHS Foundation Trust, Fulbourn, UK; 19 Forth Valley Eating Disorder Service, Stirling, UK; 20 Berkshire Eating Disorders Service, Berkshire Healthcare NHS, Bracknell, UK; 21 Talking Therapies, Berkshire Healthcare NHS, Bracknell, UK; 22 Royal Holloway University of London, London, UK

**Keywords:** Adult community eating disorders services, referral rates, commissioning guidance, workforce levels, *Ignoring the Alarms* report

## Abstract

**Aims/method:**

This national pre-pandemic survey compared demand and capacity of adult community eating disorder services (ACEDS) with NHS England (NHSE) commissioning guidance.

**Results:**

Thirteen services in England and Scotland responded (covering 10.7 million population). Between 2016–2017 and 2019–2020 mean referral rates increased by 18.8%, from 378 to 449/million population. Only 3.7% of referrals were from child and adolescent eating disorder services (CEDS-CYP), but 46% of patients were aged 18–25 and 54% were aged >25. Most ACEDS had waiting lists and rationed access. Many could not provide full medical monitoring, adapt treatment for comorbidities, offer assertive outreach or provide seamless transitions. For patient volume, the ACEDS workforce budget was 15%, compared with the NHSE workforce calculator recommendations for CEDS-CYP. Parity required £7 million investment/million population for the ACEDS.

**Clinical implications:**

This study highlights the severe pressure in ACEDS, which has increased since the COVID-19 pandemic. Substantial investment is required to ensure NHS ACEDS meet national guidance, offer evidence-based treatment, reduce risk and preventable deaths, and achieve parity with CEDS-CYP.

Eating disorders are increasing in the UK. According to the 2019 Health Survey for England, 16% of adults screened positive for an eating disorder and 4% reported significant impairment in their functioning.^1^ This represents a nearly threefold increase since the Adult Psychiatric Morbidity survey of 2007.^2^

Historically, adult community eating disorder services (ACEDS) in the UK have been under-resourced. The Parliamentary and Health Service Ombudsman (PHSO) highlighted this in a report titled *Ignoring the Alarms: How NHS Eating Disorders Services Are Failing Patients*.^3,4^ The PHSO called for parity of investment for ACEDS, to match the expansion of community services for children and young people with eating disorders (CEDS-CYP) and the NHS England (NHSE) access and waiting time standards for community CEDS-CYP treatment.^5^ Subsequently, in 2019, NHSE published commissioning guidance for adult community, day and in-patient services.^6^ As part of the long-term plan, this remains an aspirational document with no specific assessment of implementation costs.^7^ The Welsh Government published a review of eating disorder services in 2018,^8^ and the Scottish Government in 2021.^9^ These included important recommendations that are yet to be implemented.

In contrast to CEDS-CYP, there is no systematic data collection at the national level regarding access and waiting times for adults with eating disorders and there are no reliable baseline data on the size of the demand (e.g. number of referrals) and capacity (number of staff needed) for ACEDS to deliver the NHSE commissioning guidance for adults with eating disorders.^6^

## Aims and objectives

The aim of this survey was to establish a baseline of ACEDS demand (e.g. referral rates) and capacity (e.g. staffing levels) prior to the pandemic and prior to investment, as well as whether services could comply with NHSE commissioning guidance.^6^ This information, with costing implications, will help guide future commissioning and improve timely access to evidence-based treatment for adults with eating disorders.

## Method

This is a survey of ACEDs regarding demand and capacity in comparison with NHSE commissioning guidance standards.^6^

We asked participating services to report referral patterns and staffing levels for total budgeted full-time equivalent (FTE) and total actual FTE staff in post on 31 March 2020 (Supplementary Demand and Capacity Excel Survey Questions, available at https://dx.doi.org/10.1192/bjb.2023.57). We compared existing staffing levels with recommended staffing levels using the NHSE CEDS-CYP workforce calculator and Personal Social Services Research Unit (PSSRU) costs for health and social care in 2013–2014, as recommended for commissioners of adult services.^5^

An additional online survey included 41 questions on whether services could comply with the NHSE Commissioning guidance, the majority of which asked respondents to select ‘yes’, ‘partially’ or ‘not at all’, with prompts to elaborate using an open-text box. The Survey Monkey Questions are included in the Supplementary files.

### Analysis

Data were analysed with Microsoft Excel. Individual service statistics were converted to a million population to facilitate comparisons of demand (i.e. median, range and mean number of referrals) and capacity (e.g. median, range and mean total budgeted FTE and total actual FTE workforce) per million population.

Two members of the research team classified qualitative data separately for each question, and common concepts were deductively classified into a coding scheme. Responses from the online survey are summarised in Supplementary Table 1.

### Setting

There was no information available at the time of the study on the number of ACEDS in the UK. The first and last authors approached 21 services using their professional networks (Faculties of Eating Disorders, British Psychological Society Division of Clinical Psychology, and the Royal College of Psychiatrists). In Wales and Northern Ireland, there were no specialised services. In total, 13 ACEDS (12 from England and 1 from Scotland) covering a combined population of 10.7 million completed the online survey. Given that the survey was concluded on 31 March 2020 (i.e. a week after the first COVID-19 lockdown), the response rate (62%) was satisfactory. In addition, up to 8 of the 13 services (covering 7.3 million population) supplied further information on referral patterns and workforce levels.

The participating services were:

- Berkshire Eating Disorder Service
- Buckinghamshire Eating Disorder Service
- Cambridgeshire and Peterborough Adult Eating Disorder Service
- ‘Community Eating Disorder Service’ (location not specified in service name)
- Dorset Eating Disorders Service
- Gloucestershire Eating Disorders Service
- Hertfordshire Community Eating Disorder Service
- NHS Forth Valley Eating Disorder Service
- Oxford Community Eating Disorders Service
- Sheffield Eating Disorders Service
- South London & Maudsley NHS Foundation Trust Adult Eating Disorders Outpatient Service
- Surrey and Borders Partnership NHS Foundation Trust Adult Eating Disorders Service
- Wiltshire Eating Disorders Service.

Services represented a variety of demographics, including rural, urban and university populations; 62% of trusts provided in-patient care and 85% offered intensive day therapy. We provide anonymised data in this paper.

### Ethics

Individual patient consent was not needed. Each participating service registered a clinical audit with their respective quality and audit teams. The Oxford Health NHS Foundation Trust Audit Department approved the overall study.

## Results

### Demand: annual referral patterns

Only seven services (total population: 6.54 million) were able to provide annual referral data for four consecutive financial years. The mean number of referrals received increased by 18.8% between 2016–2017 and 2019–2020, from 378 (s.d. = 106) to 449 (s.d. = 111, range: 330–643) per million population. The rate of accepted referrals dropped from 94% to 87%, 88% and 84% over the 4 years.

Table 1 shows a breakdown (median, range, mean percentage) of the available data per million population for the referral sources, age and gender of referrals for 2019–2020 for six services (total population 6 million).

**Table 1:** Demand: source, age and gender of referrals to six adult community eating disorder services in 2019–2020 per million population

		Referrals, median (range)^a^	Mean % of referrals
Source of referrals	General practitioner	294 (136–467)	68.3
	Adult community mental health team	22 (18–160)	13.3
	Child and adolescent mental health services	6 (0–54)	3.7
	Self	0 (0–36)	1.6
	Acute mental health in-patient units	0(0–6)	0.5
	Other	59 (1–99)	12.6
Age range, years	18 to <25	210 (157–341)	46.3
	≥25	244 (163–340)	53.7
Gender	Male	37 (25–59)	8.2
	Female	416 (300–584)	91.7
	Other	0.3 (0–1)	0.1

a. Medians and ranges are included as the data for ‘Source of referrals’ were not normally distributed.

General practitioners (GPs) and adult community mental health teams (CMHTs) were the most common referral sources. Less than half of those referred were under 25 years of age; 92% were female.

### Capacity: workforce levels on 31 March 2020

Table 2 provides a snapshot of workforce levels on 31 March 2020 and compares the NHSE CEDS-CYP workforce calculator^5^ recommendations and associated staffing mix with the median, range and total mean of budgeted and occupied FTE staffing roles per million population for 449 referrals/year for the 13 ACEDS.

**Table 2:** Capacity: budgeted and occupied full-time equivalent (FTE) staffing roles for 13 adult community eating disorder services on 31 March 2020 compared with NHSE CEDS-CYP workforce calculator^5^ recommendations

Role (NHS pay bands)	Total budgeted FTE posts per million population, median (range)	Total actual FTE occupied posts per million population, median (range)	NHSE CEDS-CYP workforce calculator estimates of 21.5 FTE staff/100 referrals adjusted to 449 referrals/year	Percentage of ACEDS total budgeted mean FTE/NHSE CEDS-CYP workforce calculator staffing mix recommendations
Consultant psychiatrist	0.7 (0.3–1.7)	0.7 (0–1.7)	5.4	22%
Consultant psychologist	0.1 (0–0.8)	0.1 (0–0.8)		
Psychiatrists, e.g. specialty doctors, trainees	0 (0–0.7)	0.2 (0–0.9)	7.2	3%
Medical professionals: GP or physician	0	0	0.9	0%
Psychologists (8B)	No data	No data	1.3	−
Operational/team manager (8A)	0.6 (0–1.9)	0.7 (0.3–1.9)	−	−
Psychologists/ psychological therapists (7/8A)	2.5 (1.4–7.1)	2.9 (1.2–6.2)	35.9	10%
Systemic family therapists (7/8A)	0.3 (0–0.6)	0.3 (0–0.6)		
Nursing staff (6)	2.2 (1.4–8.4)	1.5 (0.9–8.3)	11.2	30%
Social workers (6)	0 (0–1)	0 (0–1)	−	−
Occupational therapists (6)	0.9 (0–1.9)	0.9 (0–1.6)	−	−
Dietitians (6)	0.9 (0.3–1.2)	0.9 (0.3–1.1)	6.7	12%
Assistant psychologists	0.7 (0–2.3)	0.8 (0–2.3)	8.1	12%
Assistant psychologists/data analysts/research assistants (4)	0 (0–0.3)	0 (0–0.3)		
Support workers (4)	0.6 (0–2.2)	0.6 (0–2.2)	−	−
Peer support workers (4)	0	0	−	−
Office managers (4)	No data	No data	3.6	16%
Administrative staff (4)	1.9 (1.4–2.5)	1.7 (0.8–2.5)	8.1	
Other	0 (0–0.8)	0 (0–0.8)	8.1	2%
Total FTE posts, median (range)	14.3 (10.3–19.2)	14.6 (9.9–17)	−	−
Total FTE posts, mean	14.3	13.9	96.5	15%

NHS, National Health Service; NHSE, NHS England; CEDS-CYP, child and adolescent eating disorder services; ACEDS, adult community eating disorder services; GP, general practitioner.

The recommended staffing for CEDS-CYP for 100 referrals per year is 21.5 FTE, at a cost of £1 559 061 (based on PSSRU unit costs for health and social care in 2013–2014).^5^ After adjusting for 449 referrals/year, the ACEDS would require 96.5 FTE, at a cost of £7 000 183, to achieve parity with CEDS-CYP. This meant that on 31 March 2020, the mean total budgeted FTE posts for the ACEDS were 15% of NHSE recommendations for CEDS-CYP, with 14% of the necessary staff in post to meet the demand and patients’ needs.

### Capacity of ACEDS to meet the NHSE guidance for commissioners and providers

Table 3 summarises survey responses on whether the 13 ACEDS had the capacity to meet the NHSE commissioning guidance for adults with eating disorders.

**Table 3:** Survey responses on whether the 13 adult community eating disorder services met the NHS England commissioning guidance for adults with eating disorders^6^

Survey statement	Able to meet standard?	
	Yes	Partially
Referrals of all presentations of eating disorders are accepted, regardless of length of illness, severity or BMI	31%	0%
Evidence-based treatment, care and support is offered for all eating disorders, including BED, ARFID and OSFED	54%	0%
The service has capacity for managing risks safely	62%	38%
The service has capacity to follow-up patients (e.g. who are not engaging, not attending appointments) and avoids inappropriate discharge	46%	54%
The prevalence of eating disorders and demand for services in the local area has been assessed using e.g. the Public Health Fingertips Tool	15%	15%
The service/trust offers intensive day-patient treatment for patients with eating disorders	85%	0%
The service received an increase in annual recurring investment over the last 5 years since 2014–2015	38%	0%
Access to care is equal regardless of whether a person presents for first time or with a long-term eating disorder	77%	23%
Individuals can self-refer to access the service, including when re-presenting at first sign of relapse	23%	8%
The service has a waiting list for treatment	92%	8%
Commissioners develop and implement local plans in collaboration with people with experience, service providers and partner agencies	54%	15%
The service has the capacity to take responsibility for outreach, follow-up and engaging with people who are reluctant to receive treatment	46%	8%
If a patient is reluctant to engage, and there is evidence of recent deterioration or severe risk, support is offered indirectly by engaging patients’ families, partners, carers or members of their support network	77%	8%
The service provides full medical monitoring (including blood tests and ECGs with same-day results)	38%	31%
The service has an agreed protocol with primary care services to ensure physical assessment and monitoring of patients	23%	62%
The service has support from acute medical care, including emergency admissions	54%	46%
The service remains the lead in providing care, working closely with in-patient staff from the start of the admission to discharge, to ensure persons receive appropriate levels of treatment	54%	46%
Intensive community treatment is offered as an alternative to in-patient treatment	38%	46%
For age-based transitions, the service works with the relevant CEDS-CYP team for a minimum of 6 months before planned transitions	38%	39%
The service has sufficient capacity to ensure seamless transition for people needing in-patient and day treatment, including admission and discharge planning, i.e. with psychological therapy and social components included	38%	46%
For geographical transitions, the service has capacity to work closely with primary care providers, ACEDS in other areas, and university mental health services to ensure seamless transitions and avoid gaps and delays in handovers of ongoing care and treatment, including for students during holiday times	38%	31%
Staff have specific training and skills to support patients with diabetes and diabulimia	23%	54%
Treatment is available and can be adapted for those who experience comorbid conditions, such as autism, substance misuse or personality disorders	38%	62%

BMI, body mass index; BED, binge-eating disorder; ARFID, avoidant/restrictive food intake disorder; OSFED, other specified feeding or eating disorder; ECG, electrocardiogram; CEDS-CYP, child and adolescent eating disorder services; ACEDS, adult community eating disorder services.

Ninety-two per cent of the services had a waiting list for treatment. More than half (7/13) of the services used a variety of criteria for prioritising patients on the waiting list (e.g. physical or psychiatric risks; severity of the eating disorder; pregnancy; discharges from day or in-patient services; geographical transitions) (Supplementary Table 1). Some services reported waiting times for treatment of up to 2 years.

Thirty-eight per cent of services (5/13) reported only a limited capacity to manage risks safely, citing factors such as a lack of staffing, long waiting lists and staff stress (Supplementary Table 1).

Fifty-four percent reported difficulties with service evaluation (e.g. insufficient staffing for collection, analysis and reporting of data/routine outcome measures; Supplementary Table 1).

## Discussion

To the best of our knowledge, this was the first large-scale survey of ACEDS demand, capacity and provision in the UK since a Royal College of Psychiatrists report on service distribution, development and training published in 2012.^10^

Annual referral rates increased by 18.8% between 2016–2017 and 2019–2020, and the mean number of referrals per million population in 2019–2020 was 449 (range: 330–643). During the same period, the rate of accepted referrals fell from 94% to 84%, reflecting system stress and increasingly stringent referral acceptance criteria. In comparison, according to a 2008 survey of child and adult eating disorder services in the UK and Ireland, only 50% of services received more than 25 referrals per year.^10^ In parallel, in-patient admissions have also increased annually,^11,12^ indicating that the current increased demand spans the full range of eating disorder severity.

On 31 March 2020, in comparison with the 2015 NHSE staffing recommendations for CEDS-CYP services,^5^ the ACEDS were only 15% funded and 14% staffed (based on the CEDS-CYP workforce calculator and the PSSRU costs for health and social care in 2013–2014) and therefore could not meet the needs of existing case-loads and annually referred patients. To achieve parity with CEDS-CYP the estimated budget of an ACEDS with 449 referrals/million population/year should have been £7 million. These figures need to be adjusted for 2023–2024 and this should also include capital investment.

The 13 ACEDS, unsurprisingly, lacked the capacity to meet several NHSE commissioning standards, including the ability to provide timely evidence-based treatments, treat the entire spectrum of eating disorders (including binge-eating disorder, avoidant/restrictive food intake disorder (ARFID) and other specified feeding or eating disorder (OSFED)), manage transitions between services and provide assertive outreach for vulnerable, hard-to-reach populations. Liaison with primary care and acute medical services, as well as the ability to provide medical monitoring, also fell short of standards. The COVID-19 pandemic has further exacerbated the long waiting lists caused by the demand exceeding capacity.^13,14^

As in the Public Administration and Constitutional Affairs Committee's follow-up on *Ignoring the Alarms* in 2019,^4^ the national picture in this survey reflects little progress since the death of Averil Hart in 2012^3,15^ and highlights the ongoing high risk in ACEDS, as well as the potential for additional avoidable deaths. Since the PHSO's first report in 2017,^3^ another 19 deaths were identified where coroners expressed concerns; 15 of these deaths were deemed avoidable.^16^ In 2023, the PHSO renewed his call for urgent improvement in adult services, stating that patients are repeatedly failed by the system and that lives are being lost owing to lack of parity between child and adult services, poor coordination between those involved in treating patients and lack of training.^17^

### Demand: annual referral patterns

The increase in annual referrals to the 13 ACEDS is comparable with the increase in eating disorder prevalence estimates reported in the 2019 Health Survey and the hospital statistics.^1,12^ It implies that only a small percentage of those who would benefit from treatment receive care. The geographical distribution of eating disorders differs according to factors such as the presence of higher education institutions and rural versus urban areas. The next adult psychiatry morbidity survey should contribute to a more accurate estimation of prevalence rates in the UK.

Even though both men and women could benefit from treatment, 92% of the referrals were of women. This is consistent with earlier studies in specialist services.^18^ According to the 2019 Health Survey, 3% of men aged 16 and older report significant impairment due to eating disorders.^1^ This indicates a substantial level of unmet need that needs to be addressed without delay.

The increasing demand for in-patient admissions^11^ may indicate that ACEDS are unable to deliver timely evidence-based therapies to prevent patients from deteriorating severely. NHSE Digital^12^ reported a fourfold rise in hospital admissions of people with primary or secondary eating disorder diagnoses between 2007–2008 and 2020–2021 (~70% were adults). In 2012, the Royal College of Psychiatrists suggested six in-patient beds per million population for the treatment of eating disorders.^10^ In contrast, the HOPE Provider Collaborative required an average of 12 in-patient beds per million population in 2018–2019; after the pandemic, this number has risen to 15.^19^ These results strengthen the case for immediate investment in ACEDS to improve access to levels comparable to those in CEDS-CYP. This could help reverse the rising trend of hospital admissions.

It is important to note that only 3.7% of referrals were CEDS-CYP transitions. This demonstrates the success of the investment in CEDS-CYP: the majority of patients do not require further treatment for ACEDS after receiving timely evidence-based treatment. The small number of adolescents who transition to ACEDS usually have persistently low weight and significant levels of complexity and comorbidity, necessitating intensive resources following transition.^20^

Approximately half of the ACEDS referrals were of people between the ages of 18 and 25. This is consistent with recent research indicating that the median age at onset of eating disorders is 18 years.^21^ There has been some minor investment in programmes for 18- to 25-year-olds in recent years, particularly first-episode rapid early intervention for eating disorders (FREED).^22^ Since the beginning of the pandemic, however, referrals to FREED services in England have increased by a factor of 1.4 (compared with a referral increase of 1.2 to CEDS-CYP), with a relative increase in anorexia nervosa cases among these referrals.^23^ Given that more than half of ACEDS patients are older than 25, it is evident that investments in FREED will not be sufficient to meet the needs of the patient population. In addition, FREED is not yet available outside of England and cannot be implemented on a large scale until substantial investments are made.

### Workforce levels on 31 March 2020

This survey confirmed geographical inequity of services, with wide variations in staffing levels and roles. For example, the budgeted posts for consultant psychiatrists in the ACEDS in our study ranged from 0.3 to 1.7 FTE/million population and those for psychologists/psychological therapists ranged from 1.4 to 7.1 FTE/million population. The budgeted FTE/million for the largest service (19.2) was nearly double that of the least resourced service (10.3). The mean case-load for psychiatrists was approximately 500/year. This is incompatible with the safe management of physical and mental comorbidities, and results in poor staff recruitment and retention. Similarly, the CEDS-CYP workforce calculator suggested 36 FTE psychologists/therapists for 449 referrals annually, but the services in our survey had a mean of 3.6 psychologists/therapists in posts. With this staffing level, only a small fraction of patients can receive the NICE-recommended treatment. In addition, case-loads will increase dramatically once services accept the full spectrum of eating disorders (in terms of diagnosis and severity).

The mean total number of budgeted FTE posts in our ACEDS was 14.3/million population. By comparison, the CEDS-CYP workforce calculator recommended 96.5 FTE posts to meet the mean demand of 449 referrals/million population. This means that, prior to the pandemic, these ACEDS were only 15% funded and 14% staffed to meet demand. Such grossly inadequate resources endanger patient safety and may contribute to staff burnout across the UK. Furthermore, the situation has deteriorated since the pandemic.^24^ Given that evidence-based treatment benefits two-thirds of patients,^25^ investment in services would be cost-effective, as it would help reduce the number of people developing a chronic condition.

### Capacity of ACEDS to meet the NHSE guidance for commissioners and providers

The aim of the NHSE commissioning guidance^6^ was to provide guidance on the most effective models of service delivery to enhance access to treatment and support for adults with eating disorders. Owing to the annual increase in referrals and inadequate staffing, most services in our survey were unable to adhere to all the standards of good practice.

There was a waiting list for treatment for all except one service, and rationing strategies were implemented (e.g. body mass index, illness severity, exclusion of some eating disorder diagnoses). This had an impact on patient safety throughout the care pathway. Long waiting lists for those with potentially life-threatening eating disorders requiring hospital treatment have been reported, for example, by the HOPE Provider Collaborative.^19^ There were additional commissioning challenges for individuals with complex problems and comorbidities (e.g. those with personality disorders or autism), which have worsened since the pandemic.^13,19^ Excessive waiting times for admission drive up the risks in ACEDS and are a contributing factor to an increasing number of emergency medical admissions prior to specialist eating disorder admissions. It is also likely that the risk levels in ACEDS could contribute to recruitment and retention difficulties, which further exacerbate the situation.

Only 38% of services had the capacity to ensure seamless transition and treatment from day/in-patient units to the community. Research suggests that the risk of relapse is highest in the first 60–90 days after discharge from hospital, and intensive treatment is necessary to help the patient achieve the best outcome.^26–30^ Without the ability to provide seamless transitions between in-patient and out-patient settings, more than 50% of individuals relapse within a year of hospital discharge; this number could be reduced to 15% with integrated treatment, and the number of high-risk patients could be reduced over the medium term.^27^

Eating disorders are associated with high levels of comorbidity and risk of mortality,^31–33^ and the effective management of these requires sufficient highly skilled staff. In our survey, 62% of services reported only a limited ability to modify treatment for patients with comorbid conditions such as autism, substance use disorders or personality disorders. Most teams lacked the necessary training to help patients with diabetes. This is a previously ignored patient population with a high risk of irreversible consequences and poor experience of services.^34–37^

Risk management had significant shortcomings. Only around 40% of services could provide complete medical monitoring and had good links with acute hospitals.

Responsibility for outreach, follow-up and engagement with patients who do not engage in treatment lies with the ACEDS,^6^ yet only 50% of services met the guideline. Unfortunately, as the severity of a patient's illness increases, the likelihood of seeking and accepting help decreases significantly, so the inability to provide assertive outreach further increases the risk of deterioration.

Students who leave home are an especially vulnerable group as poor transitions between services have a significant negative impact on their ability to achieve academic success or benefit from broader aspects of university life.^6,25^ NICE guidelines^25^ recommend well-coordinated care for students who require help in different locations at different times of the year. Yet, only 38% of the ACEDS were able to ensure seamless transitions between home and university services (including during holidays). Failure to provide safe transitions between services is associated with increased risk, poor care experiences, disengagement, poor treatment outcomes and avoidable admissions.^25,38,39^

The co-production and co-delivery of ACEDS is essential,^40^ yet co-production is often not costed or adequately remunerated for people with lived experience. In the future, this issue will need to be addressed and funded co-production has to be included in the staffing mix.

Our survey showed that even before the pandemic ACEDS were severely underfunded and understaffed, leaving services unable to meet rising demand. This had a major impact on their ability to provide timely evidence-based treatments and manage risks safely for adult patients. Many of the NHSE Commissioning guidelines for ACEDS were not met. Given that the median onset age for developing an eating disorder is 18, and that only 3.7% of patients are referred from CEDS-CYP, investment in CEDS-CYP, while beneficial, cannot reverse the rising trend of patients over the age of 18 who require treatment. With the continued rise in eating disorders since the pandemic, ACEDS requires significant new funding to prevent further avoidable suffering and deaths, and to maintain its reputation for providing safe and effective NHS services without discrimination to people of all ages.

### Strengths and limitations

This paper addresses an important gap in the literature regarding the status of ACEDS in England and Scotland and aimed to establish referral rates, staffing levels and ability to meet commissioning guidance prior to the COVID-19 pandemic. The large data-set contained information for a population of 6–10 million people (17.2% of the population of England and Scotland). We therefore consider the data to be representative of the majority of ACEDS in England and, to a lesser degree, Scotland (where only one service participated). Additional strengths of the paper are that staffing and costing implications to address referral rates are discussed.

There are several limitations to this study. At the time of the study 21 services were approached as no data were available on the total number of ACEDS in the UK. Since then the FREED study has identified 54 NHS trusts in England with ACEDS (U. Schmidt, personal communication, 2023). The 13 services from which data were collected may therefore not be representative, and were a convenience sample based on personal connections, rather than systematic attempts to contact all ACEDS in the UK. Furthermore, the sample of ACEDS were relatively well-known and active in eating disorder research, publication and conferences. This might have resulted in a more favourable impression of service provision and an underestimation of the extent of unmet need (i.e. if lesser-known services had been included). It would be interesting to learn how the results compare with service provision, demand and capacity in Wales and Northern Ireland.

Some services were unable to provide data on every aspect of the survey. Unlike CEDS-CYP, ACEDS are not required to record information about access and waiting times. Data on ethnicity and other protected characteristics, comorbidities and outcomes were unavailable for the majority of services. The pandemic not only affected data collection, but also contributed to a worsening of the demand and capacity crisis highlighted by this survey. Finally, the differing costs between NHS and private/independent sector services, and the consequences of a mixed economy for costs and joined-up care, fell outside the scope of the survey.

### Recommendations

Based on our findings the main recommendations are as follows:

- The NHS should commission cost estimates to ensure that national guidelines (e.g., NICE,^25^ NHSE commissioning guidance,^6^ Welsh^8^ and Scottish^9^ reviews) are implemented and funding should be allocated to achieve parity of access to timely evidence-based treatment across the age range.
- As a result of an increase in the number and severity of eating disorder presentations related to the COVID-19 pandemic, the government should provide emergency funding to meet the urgent needs of eating disorder patients and services.
- Access and waiting time for eating disorder services should be monitored across the age range as part of a national audit of services.
- ACEDS should be co-designed, co-produced and co-delivered in collaboration with adequately remunerated and diverse groups of patients and carers who have lived experience with a variety of eating disorders.
- Staff must be sufficiently trained to deliver high-quality ACEDS and modify therapies for comorbid illnesses, such as autism, substance misuse, personality disorders and diabetes.
- Retention of experienced, highly trained staff is a priority to ensure that recruitment and training occur alongside the provision of service excellence.
- All eating disorder services should offer training placements for relevant disciplines, such as psychiatry, psychology, nursing, dieticians.
- Men and individuals from ethnic minorities, as well as other underserved populations, must be the focus of new funding for treatment developments.
- Transitions (particularly for students and transfers between in-patient/day patient and community services) should be seamless for all patients across the care pathway to help them achieve the best outcomes.
- The needs, obstacles and adaptations required in the treatment of eating disorders in rural populations require further exploration.
- ACEDS require dedicated resources for research and audits to improve treatment outcomes and service development.
- A list should be produced of all ACEDS in the UK. The study should then be replicated to include all ACEDS to remove any potential participant bias.

## Supporting information

Viljoen et al. supplementary material 1 Viljoen et al. supplementary material

Viljoen et al. supplementary material 2 Viljoen et al. supplementary material

Viljoen et al. supplementary material 3 Viljoen et al. supplementary material

Viljoen et al. supplementary material 4 Viljoen et al. supplementary material

## Data Availability

The anonymised data that support the findings of this study are available from the corresponding author, D.V., on reasonable request.

## About the authors

**David Viljoen** was a consultant clinical psychologist with Cotswold House, Adult Community Eating Disorder Service Oxford, Oxford Health NHS Foundation Trust (OHFT), UK, and is now a consultant clinical psychologist at Ellern Mede Group, London, UK. **Emily King** was an assistant psychologist with Adult Community Eating Disorder Service Oxford, OHFT, UK, and is now a senior assistant psychologist in the Support to Recovery in Disordered Eating Service for 18–25s at Central and North West London NHS Foundation Trust, London, UK. **Sophie Harris** was an assistant psychologist with Adult Community Eating Disorder Service Oxford, OHFT, UK, and is now a trainee clinical psychologist at Royal Holloway University of London, London, UK. **Jonathan Hollyman** was an assistant psychologist with Adult Community Eating Disorder Service Oxford, OHFT, UK, and is now a trainee clinical psychologist with Salomons Institute for Applied Psychology, Canterbury Christ Church University, Canterbury, UK. **Kate Costello** was an Assistant Psychologist with Adult Community Eating Disorder Service Oxford, OHFT, UK, and is now a trainee clinical psychologist at the School of Health in Social Science Clinical Psychology, University of Edinburgh, Edinburgh, UK. **Eimear Galvin** was an assistant psychologist with OHFT, UK, and is now a trainee clinical psychologist with Oxford Institute of Clinical Psychology Training and Research, Oxford University and OHFT, Oxford, UK Oxford, UK. **Melissa Stock** was an assistant psychologist with Hertfordshire Community Eating Disorder Service, Hertfordshire Partnership University NHS Foundation Trust, UK, and is now a trainee clinical psychologist with Oxford Institute of Clinical Psychology Training and Research, Oxford University and OHFT, Oxford, UK. **Ulrike Schmidt** is a Professor of Eating Disorders at the Institute of Psychiatry, Psychology and Neuroscience, King's College London, UK. **James Downs** is a patient representative with the Royal College of Psychiatrists, London, UK. **Murali Sekar** is a medical director and consultant psychiatrist in eating disorders with the Priory Hospital, Chelmsford, UK. **Ciaran Newell** was a nurse consultant with the Dorset Eating Disorders Service, Dorset Healthcare University NHS Foundation Trust, and is now an independent nurse consultant and visiting Fellow at Bournemouth University, Poole, UK. **Sam Clark-Stone** is a lead clinician with the Eating Disorders Service, Gloucestershire Health and Care NHS Foundation Trust, Brockworth, UK. **Amy Wicksteed** is a lead clinical psychologist with Sheffield Eating Disorder Service, Sheffield Health and Social Care NHS Foundation Trust, Sheffield, UK. **Caroline Foster** is a consultant clinical psychologist with Surrey and Borders Adult Eating Disorder Service, Surrey and Borders Partnership NHS Foundation Trust, UK. **Francesca Battisti** is a consultant psychiatrist with the Wiltshire Community Eating Disorders Service, OHFT, Oxford, UK. **Laura Williams** was an assistant psychologist with Surrey and Borders Adult Eating Disorder Service, Surrey and Borders Partnership NHS Foundation Trust, Leatherhead, UK, and is now a trainee clinical psychologist at the School of Health in Social Science Clinical Psychology, University of Edinburgh, Edinburgh, UK. **Roshan Jones** was a clinical psychologist with Oxford Adult Community Eating Disorder Service, OHFT, UK, and is now a clinical psychologist with Oxford Child and Adolescent Eating Disorder Service, OHFT, Oxford, UK. **Sarah Beglin** is a consultant clinical psychologist with Adult Eating Disorder Service Cambridgeshire and Peterborough, Cambridgeshire and Peterborough NHS Foundation Trust, Fulbourn, UK. **Stephen Anderson** is a consultant psychiatrist with NHS Forth Valley Eating Disorder Service, Stirling, UK. **Thuthirna Jebarsan** was an assistant psychologist with Berkshire Eating Disorders Service, Berkshire Healthcare NHS Foundation Trust (BHFT), and is now a psychological wellbeing practitioner with Talking Therapies at BHFT, Bracknell, UK. **Viviane Ghuys** is a principal clinical psychologist with Berkshire Eating Disorders Service, BHFT, Bracknell, UK. **Agnes Ayton** is a consultant psychiatrist with Cotswold House, OHFT, Oxford, UK.
