# Supplementary material for: The alarms should no longer be ignored: survey of the demand, capacity and provision of adult community eating disorder services in England and Scotland before COVID-19
Source: BJPsych Bull. 2024 Aug;48(4):212–20. doi: 10.1192/bjb.2023.57 (PMC11543318; doi:10.1192/bjb.2023.57)
Supplement: Viljoen et al. supplementary material 2 — Viljoen et al. supplementary material [file S2056469423000578sup002.docx]

|  |
| --- |
|  |
| **Survey Monkey Questions (in Microsoft Word Format)** |

Thank you for participating in this multi-site audit looking at demand and capacity trends across the UK. All data will be kept confidential and our aim is to publish anonymous data to inform future commissioning.

Every service can only use a single device (i.e. laptop/computer) as a second device would create a second response for your service.

**Please answer the following questions and whether your service has the capacity to comply with the 2019 NHSE guidelines for commissioners and providers:**

1. What is the name of your community adult eating disorder service?

2. Referrals of all presentations of eating disorders are accepted, regardless of length of illness, weight, severity or BMI

Yes

No (Please indicate any of your exclusion criteria, such as only treating moderate or severe eating disorders; not treating ARFID or comorbidities)

3. Evidence-based treatment, care and support is offered for all eating disorders, including BED, ARFID and OSFED

Yes

No (Please elaborate)

4. Consultation is offered to other services

Yes

No

Partial (Please specify)

5. The service has the capacity for managing risks safely

Yes

No

Partial (Please elaborate on any factors that impact on the service's ability to manage the risks of patients with ED safely)

6. The service has the capacity to follow-up patients (e.g. who are not engaging, not attending appointments) and avoids inappropriate discharge

Yes

No

Partial (Please elaborate)

7. Clinical supervision is delivered to professionals as per national guidelines

Yes

No

Partial (Please elaborate)

8. The prevalence of eating disorders, and demand for services in your local area has been assessed (using e.g. the Public Health Fingertips Tool)

Yes

No

Partial (Please elaborate)

9. Routine outcome measures (ROMS) are used in collaboration with patients to support their treatment and recovery

Yes

No

Partial (Please elaborate)

10. Does your service/Trust offer intensive day patient treatment for patients with eating disorders?

No

Yes (Please indicate number of spaces and opening times)

11. Does your service/Trust offer inpatient treatment for eating disorders?

No

Yes (Please indicate number of beds)

12. What is the age range of your service?

<18

18-25

>25

18 - 65

18 - no upper age limit

Lifespan (all ages)

Other (Please specify)

13. What is the total population (all ages) of the catchment area associated with your service?

14. Please indicate which psychological treatment options your service offers to patients with Anorexia Nervosa:

CBT-ED

MANTRA

SSCM

FPT

Other (Please specify)

15. Please indicate which psychological treatment options your service offers to patients with Bulimia Nervosa:

Guided Self-Help

CBT-ED (Individual)

Other (Please specify)

16. Please indicate which psychological treatment options your service offers to patients with Binge-Eating Disorder:

Guided Self-Help

CBT-ED (Groups)

CBT-ED (Individual)

Other (Please specify)

17. Please indicate the total number of sites from which your service operates:

18. Please indicate what the total annual budget (including medical staffing) is in £ for your service for 2019/20?

19. Has there been an increase in annual recurring investment in the service over the last 5 years (i.e. since 2014/15)?

No

Yes (Please elaborate and provide figures in £)

20. Has there been an increase in annual recurring investment in the service during the previous 5-10 years (i.e. between 2009/2010 and 2014/2019)?

No

Yes (Please elaborate and provide figures in £)

21. How much non-recurring annual funding (e.g. one-off investments to clear waiting lists) has been invested in your adult community eating disorders service between 2014/15 and 2019/20?

22. Access to care is equal regardless of whether a person is presenting for the first time or with a long-term eating disorder

Yes

No

Partial (Please elaborate)

23. Individuals can self-refer to access your service (including when re-presenting at the first sign of a relapse)

Yes

No

Partial (Please elaborate)

24. According to the guideline, people should 'receive treatment, care and support as soon as possible'. Does your service have a waiting list for treatment? If yes, do you use any criteria to prioritise certain patients on the waiting list for treatment?

No

Yes (Please specify or describe any criteria that you use to prioritise patients)

Severity of the Eating Disorders (e.g. DSM-5 criteria for extreme eating disorders - please describe below)

Psychiatric Risk (e.g. suicide or self-harm - please describe below)

Physical Risk (e.g. diabetes, low BMI - please describe below)

Pregnant women and women with children under 1 years

Onset of the eating disorder less than three years

Discharge from day and inpatient services

Members from the armed forces and veterans

Patients who transition geographically (e.g. students or handover requests from other eating disorder services)

We don't use any criteria to prioritise patients on the waiting list

Other (Please specify or provide more details)

25. Registration with a GP is an essential criterion for access to your service

Yes

No

26. Care can be provided using digital technologies (e.g. SKYPE/FaceTime) if required

Yes

No

Partial (Please elaborate)

27. Commissioners develop and implement local plans in collaboration with people with experience, service providers and partner agencies.

Yes

No

Partial (Please elaborate)

28. The Community Eating Disorder Service has the capacity to take responsibility for outreach, follow-up and engaging with people who are reluctant to receive treatment.

Yes

No

Partial (Please elaborate)

29. If a patient is reluctant to engage, and there is evidence of recent deterioration or severe risk, support is offered indirectly by engaging patients’ families, partners, carers or members of their support network

Yes

No

Partial (Please elaborate)

Medical Monitoring

Please indicate whether your service has the capacity to comply with the following guidelines regarding medical monitoring:

30. Your service is able to offer full medical assessment, including blood tests and ECGs (with same-day results)

Yes

No

Partial (Please elaborate)

31. Does the service have support from acute medical care (including emergency admissions)

Yes

No

Partial (Please elaborate on any interface difficulties with acute medical care)

32. Does the service have an agreed protocol with primary care services to ensure physical assessment and monitoring of patients?

Yes

No

Partial (Please elaborate on any areas of difficulty or lack of clarity regarding responsibilities)

33. The Community Eating Disorder Service remains the lead in providing care, working closely with inpatient staff from the start of the admission to discharge, to ensure the person receives the appropriate level of treatment

Yes

No

Partial (Please elaborate)

34. Intensive community treatment is offered as an alternative to inpatient treatment

Yes

No

Partial (Please elaborate)

35. For age-based transitions, your service works with the relevant CAMHS team for a minimum of 6 months before the planned transition

Yes

No

Partial (Please elaborate)

36. The service has sufficient capacity to ensure seamless transition for people needing inpatient and day treatment, and this includes admission and discharge planning (i.e. with psychological therapy and social components included)

Yes

No

Partial (Please elaborate)

37. For geographical transitions, your service has the capacity to work closely with primary care providers, community ED services in other areas, and university mental health services to ensure seamless transitions and avoid any gaps and delays in any handovers of ongoing care and treatment (including for students during holiday times).

Yes

No

Partial (Please elaborate)

38. Staff in your service have specific training and skills to support people with diabetes and ‘diabulimia’

Yes

No

Partial (Please elaborate)

39. Treatment is available and can be adapted for those who may experience comorbid conditions, such as autism, substance misuse or personality disorders.

Yes

No

Partial (Please elaborate)

40. Please feel free to add any additional comments regarding demand and capacity issues in your service.

41. Did this survey highlight any difficulties that your service experience regarding service data/evaluation (e.g. staffing capacity for data collection and analysis, reporting of data/ROMS, etc.)?

No

Yes

If yes, please specify
