# Supplementary material for: The alarms should no longer be ignored: survey of the demand, capacity and provision of adult community eating disorder services in England and Scotland before COVID-19
Source: BJPsych Bull. 2024 Aug;48(4):212–20. doi: 10.1192/bjb.2023.57 (PMC11543318; doi:10.1192/bjb.2023.57)
Supplement: Viljoen et al. supplementary material 3 — Viljoen et al. supplementary material [file S2056469423000578sup003.docx]

| **Supplementary Table 1**   Table 1 below summarises the responses from the online survey on Survey Monkey.  **Supplementary Table 1: Online Survey Responses** | | | | |
| --- | --- | --- | --- | --- |
| Question | Yes (%) | No (%) | Partial (%) | Missing data (%) |
| Referrals of all presentations of eating disorders are accepted, regardless of length of illness, weight, severity or BMI? | N = 4 (30.77%) | N = 9 (69.23%)  *Mild symptoms excluded 5  *ARFID excluded 4  *BED excluded 1  *ARFID if no physical risks excluded 2 | N/A | N = 0 |
| Is evidence-based treatment, care and support offered for all eating disorders, including BED, ARFID and OSFED? | N = 7 (53.85%) | N = 6 (46.15%)  *Do not treat ARFID 5  *Do not treat ARFID if no physical risks 1 | N/A | N = 0 |
| Consultation is offered to other services? | N = 12 (92.30%) | N/A | N =1 (7.70%) | N = 0 |
| Does the service have capacity for managing risks safely? | N = 8 (61.54%) | N = 0 | N = 5 (38.46%)  *Lack of staffing & resources 4  *Waiting lists 2  *Staff stress 1  *Funding barriers 1  *Improvements being made 2 | N = 0 |
| The service has the capacity to follow-up patients (e.g. who are not engaging, not attending appointments) and avoids inappropriate discharge? | N = 6 (46.15%) | N/A | N = 7 (53.85%)  *Dependent on resources 3  *No outreach service 1  *Dependent on risk 3 | N = 0 |
| Clinical supervision is delivered to professionals as per national guidelines? | N = 13 (100%) | N/A | N/A | N = 0 |
| The prevalence of eating disorders, and demand for services in your local area has been assessed (using e.g. the Public Health Fingertips Tool)? | N = 2 (15.38%) | N = 8 (61.54%) | N = 2 (15.38%)  *Assessed using a different tool 2 | N = 1 (7.70%) |
| Routine Outcome Measures (ROMS) are used in collaboration with patients to support their treatment and recovery? | N = 11 (84.62%) | N/A | N = 2 (15.38%) | N = 0 |
| Does your service/Trust offer intensive day patient treatment for patients with eating disorders? | N = 11 (84.62%)  *report range/categories? | N = 2 (15.38%) | N/A | N = 0 |
| Does your service/Trust offer inpatient treatment for eating disorders? | N = 8 (61.54%) | N = 5 (15.38%) | N/A | N = 0 |
| Increase in annual recurring investment over the last 5 years (since 2014/2015)? | N = 5 (38.46%)  *For specific staff recruitment 3  *To help meet demand & improve access 1  *To manage risk 1 | N = 6 (46.15%) | N/A | N = 2 (15.38%) |
| Has there been an increase in annual recurring investment in the service during the previous 5-10 years (i.e. between 2009/2010 and 2014/2019)? | N = 7 (53.85%)  *Expansion of catchment 1  *Expansion of services 3  *To hire more staff 2 | N = 4 (30.77%) | N/A | N = 2 (15.38%) |
| Equal access to care regardless of number of referrals to services | N = 10 (76.92%) | N = 0 | N = 3 (23.08%)  *Priority to recent onset 3  *Priority to adolescents 2 | N = 0 |
| Individuals can self-refer to access service | N = 3 (23.08%) | N = 9 (69.23%) | N = 1 (7.70%)  *For under 18 y/o 1 | N = 0 |
| Services that use a waiting list for treatment (prioritisation of WL) | N = 10 (76.92%)  *Physical risk N = 12  Severity of the ED N = 10  Women and women with.. N = 10  Discharge from day/inpatient… N = 10  Transition geographically N = 7  Psychiatric risk N= 7  Onset of ED <3 years N = 6  Members of armed forces N = 3  History of AN N = 1  Length of time on waiting list N = 1  Age<19 N= 1 | N = 1 (7.70%) | N = 1 (7.70%) | N = 1 (7.70%) |
| What is the age range of your service? | N = 13 (100%)  Lifespan N. 3 (23.08%)  18+ (no upper age limit) N = 10 (76.92%) | N = 0 | N/A | N = 0 |
| Please indicate which psychological treatment options your service offers to patients with Anorexia Nervosa | N = 13 (100%)  CBT-ED N= 12 (92.31%)  MANTRA N = 12 (92.31%)  SSCM N = 10 (76.92%)  FPT N = 4 (30.77%)  DBT informed N = 4 (30.77%)  Family-based therapy N = 3 (23.08%)  CAT N = 3 (23.08%)  Integrative therapy N = 2 (15.38%)  Interpersonal psychotherapy N = 1 (7.70%)  Radically Open DBT N = 1 (7.70%) | N = 0 | N/A | N = 0 |
| Please indicate which psychological treatment options your service offers to patients with Bulimia Nervosa | GSH N = 10 (76.92%)  CBT-ED (individual) N = 12 (92.31%)  CAT N = 4 (30.77%)  Interpersonal psychotherapy N = 3 (23.08%)  CBT-T N = 2 (15.38%)  DBT Skills N = 2 (15.38%)  FPT N = 1 (7.70%)  Formulation-based therapy N = 1 (7.70%)  Family-based therapy N = 1 (7.70%)  Integrative therapy N = 1 (7.70%) | N = 0 | N/A | N = 0 |
| Please indicate which psychological treatment options your service offers to patients with Binge Eating Disorder | GSH N = 9 (69.23%)  CBT-ED (group) N = 4 (30.77%)  CBT-ED (individual) N = 7 (53.85%)  CAT N = 2 (15.38%)  DBT skills N = 2 (15.38%)  Formulation-based therapy N = 1 (7.70%)  Integrative therapy N = 1 (7.70%)  Interpersonal psychotherapy N = 1 (7.70%) | N = 0 | N/A | N = 0 |
| Registration with a GP is an essential criterion for access to your service | N = 11 (84.62%) | N = 1 (7.70%) | N/A | N = 1 (7.70%) |
| Care can be provided using digital technologies (e.g. Skype/FaceTime) if required | N = 8 (61.54%) | N/A | N = 4 (30.77%) | N = 1 (7.70%) |
| Commissioners develop and implement local plans in collaboration with people with experience, service providers and partner agencies. | N = 7 (53.85%) | N = 3 (23.08%) | N = 2 (15.38%)  *More collaboration wanted 1  *Not across all of catchment 1 | N = 1 (7.70%) |
| The Community Eating Disorder Service has the capacity to take responsibility for outreach, follow-up and engaging with people who are reluctant to receive treatment. | N = 6 (46.15%) | N = 6 (46.15%) | N = 1 (7.70%)  *Limited by capacity 5  *Only when risks are high 3 | N = 0 |
| Services that have the capacity to take on outreach, follow-up and engagement with those reluctant for treatment - support is offered indirectly by engaging patients, families, partners, carers or members of their support network | N = 10 (76.92%) | N = 2 (15.38%) | N = 1 (7.70%)  *Dependent on circumstances 1 | N = 0 |
| Services able to provide full medical monitoring, including blood tests and ECGs (with same-day results) | N = 5 (38.46%) | N = 4 (30.77%) | N = 4 (30.77%)  *Rely on other healthcare resources 3  *Only on certain days/times 1 | N = 0 |
| Services have an agreed protocol with primary care services to ensure physical assessment and monitoring of patients | N = 3 (23.08%) | N = 2 (15.38%) | N = 8 (61.54%)  *MaRSiPAN guidance used 2  *GP involvement 4   - Resistance and refusal from GP’s 3   *Process in development 3 | N = 0 |
| The Community Eating Disorder Service remains the lead in providing care, working closely with inpatient staff from the start of the admission to discharge, to ensure the person receives the appropriate level of treatment | N = 7 (53.85%) | N/A | N = 6 (46.15%) | N = 0 |
| Services that have support from acute medical care | N = 7 (53.85%) | N = 0 | N = 6 (46.15%)  *Under development 1  *Varying support 3  *No formal agreement 1 | N = 0 |
| Intensive community treatment is offered as an alternative to inpatient treatment | N = 5 (38.46%) | N = 2 (15.38%) | N = 6 (46.15%) | N = 0 |
| Do services work with CAMHS for a minimum of 6 months prior to transition? | N = 5 (38.46%) | N = 3 (23.08%) | N = 5 (38.46%)  *NA all age service 2  *Timeline unclear/not adhered to *2/3  *If contacted by CAMHS 1 | N = 0 |
| Do services have sufficient capacity to ensure seamless transition from inpatient and day treatment? | N = 5 (38.46%) | N = 2 (15.38%) | N = 6 (46.15%)  *No capacity  *Capacity for inpatient and day care  *Social components unsure  *No day patient treatment  *Offers some transition from in-patient to out-patient  *Not always possible  *Follow-up by clinician provided seamlessly  *Psychological therapy not seamless/provided | N = 0 |
| For geographical transitions, your service has the capacity to work closely with primary care providers, community ED services in other areas, and university mental health services to ensure seamless transitions and avoid any gaps and delays in any handovers of ongoing care and treatment (including for students during holiday times). | N = 5 (38.46%) | N = 4 (30.77%) | N = 4 (30.77%)  *Capacity for seamless transition 1  *Other services unable to work with capacity 1  *Insufficient capacity for rapid treatment 1  *Not always possible 2 | N = 0 |
| Do staff have specific training and skills to support patients with diabetes and diabulimia? | N = 3 (23.08%) | N = 2 (15.38%) | N = 7 (53.85%)  *Some staff members have skills and training 5  *Work closely with diabetes team 2  *Pathway with diabetes team in development 1 | N = 1 (7.70%) |
| Treatment is available and can be adapted for those who may experience comorbid conditions, such as autism, substance misuse or personality disorders. | N = 5 (38.46%) | N/A | N = 8 (61.54%) | N = 0 |
| Did this survey highlight any difficulties that your service experience regarding service data/evaluation (e.g. staffing capacity of data collection and analysis, reporting f data/ROMS, etc.)? | N = 7 (53.85%) | N = 5 (38.45%) | N/A | N = 1 (7.70%) |
| *Codes obtained from content analysis when asked to elaborate on answers. N/A = not included in survey. | | | | |
